# Do socioeconomic inequalities in mortality vary between different Spanish cities? a pooled cross-sectional analysis

**DOI:** 10.1186/1471-2458-13-480

**Published:** 2013-05-16

**Authors:** Miguel A Martinez-Beneito, Oscar Zurriaga, Paloma Botella-Rocamora, Marc Marí-Dell'Olmo, Andreu Nolasco, Joaquín Moncho, Antonio Daponte, M Felicitas Domínguez-Berjón, Ana Gandarillas, Carmen Martos, Imanol Montoya, Pablo Sánchez-Villegas, Margarita Taracido, Carme Borrell

**Affiliations:** 1 Centro Superior de Investigación en Salud Pública, Av. Cataluña, 21, 46020 Valencia, Spain; 2 Ciber de Epidemiología y Salud Pública-CIBERESP, Instituto de Salud Carlos III- Melchor Fernández Almagro, 3-5, 28029 Madrid, Spain; 3 Dirección General de Salud Pública. Conselleria de Sanitat, Generalitat Valenciana, Av. Cataluña, 21, Valencia, 46020, Spain; 4 Departamento de Ciencias Físicas, Matemáticas y de la Computación, Universidad CEU-Cardenal Herrera, C/ San Bartolomé, 55, Alfara del Patriarca, 46115, Spain; 5 Agència de Salut Pública de Barcelona, Barcelona, Spain; 6 Institut de Recerca Biomèdica Sant Pau (IIB Sant Pau), Barcelona, Spain; 7 Unidad de Investigación en Análisis de la Mortalidad y Estadísticas Sanitarias. Departamento de Enfermería Comunitaria, Medicina Preventiva y Salud Pública e Historia de la Ciencia, Universidad de Alicante, Campus de San Vicente del Raspeig s/n. Apartado 99, Alicante, 03080, Spain; 8 Observatorio de Salud y Medio Ambiente de Andalucía (OSMAN). Escuela Andaluza de Salud Pública, Campus Universitario de Cartuja, Cuesta del Observatorio, 4. Ap. Correos 2070, Granada, 18080, Spain; 9 Subdirección de Promoción de la Salud y Prevención. Dirección General de Atención Primaria, Consejería de Sanidad. Comunidad de Madrid, C/ Julián Camarillo nº 4B, 2ª planta, Madrid, 28037, Spain; 10 Departamento de Sanidad del Gobierno Vasco, Alava 45, Vitoria-Gasteiz, Álava, 01001, Spain; 11 Departamento de Medicina Preventiva y Salud Pública, Universidad de Santiago de Compostela, c/ San Francisco s/n, Santiago de Compostela, 15782, Spain; 12 Universitat Pompeu Fabra, Barcelona, Spain

**Keywords:** Deprivation, Mortality, Urban areas, Pooled cross-sectional analysis, Meta-analysis, Spain

## Abstract

**Background:**

The relationship between deprivation and mortality in urban settings is well established. This relationship has been found for several causes of death in Spanish cities in independent analyses (the MEDEA project). However, no joint analysis which pools the strength of this relationship across several cities has ever been undertaken. Such an analysis would determine, if appropriate, a joint relationship by linking the associations found.

**Methods:**

A pooled cross-sectional analysis of the data from the MEDEA project has been carried out for each of the causes of death studied. Specifically, a meta-analysis has been carried out to pool the relative risks in eleven Spanish cities. Different deprivation-mortality relationships across the cities are considered in the analysis (fixed and random effects models). The size of the cities is also considered as a possible factor explaining differences between cities.

**Results:**

Twenty studies have been carried out for different combinations of sex and causes of death. For nine of them (men: prostate cancer, diabetes, mental illnesses, Alzheimer’s disease, cerebrovascular disease; women: diabetes, mental illnesses, respiratory diseases, cirrhosis) no differences were found between cities in the effect of deprivation on mortality; in four cases (men: respiratory diseases, all causes of mortality; women: breast cancer, Alzheimer’s disease) differences not associated with the size of the city have been determined; in two cases (men: cirrhosis; women: lung cancer) differences strictly linked to the size of the city have been determined, and in five cases (men: lung cancer, ischaemic heart disease; women: ischaemic heart disease, cerebrovascular diseases, all causes of mortality) both kinds of differences have been found. Except for lung cancer in women, every significant relationship between deprivation and mortality goes in the same direction: deprivation increases mortality. Variability in the relative risks across cities was found for general mortality for both sexes.

**Conclusions:**

This study provides a general overview of the relationship between deprivation and mortality for a sample of large Spanish cities combined. This joint study allows the exploration of and, if appropriate, the quantification of the variability in that relationship for the set of cities considered.

## Background

It is well known that there is a very strong relationship between deprivation and a wide variety of causes of mortality. At a small area level [1], it has been shown that areas with high deprivation scores tend to have higher mortality than those with lower deprivation scores, but the relationship between deprivation and mortality for specific causes of death is not the same for all causes of death [2] or for all cities.

Deprivation not only means a lack of income. It also includes material and social disadvantages: on the one hand, material aspects such as diet, clothing, housing, home facilities, environment, location and work, and on the other hand, social issues such as rights in relation to employment, family activities, community relationships, participation in social institutions, recreation and education [3]. Deprivation is a relative measure where standards are defined in relation to social norms or expectations.

MEDEA (Socioeconomic and environmental inequalities in mortality in small areas in Spanish cities), a Spanish research project, studied the geographical distribution of mortality for a number of causes during the period 1996–2003 in eleven of the largest cities in Spain: Alicante, Barcelona, Bilbao, Castellón, Córdoba, Madrid, Málaga, Sevilla, Valencia, Vigo and Zaragoza, with populations ranging from 147,667 (Castellón) inhabitants to 2,938,723 (Madrid). This project studied mortality at census tract level, a small-area administrative unit with 1,212 persons per tract, on average, which allows a detailed and accurate description of the geographical variability of mortality. Specific statistical methods were necessary to estimate the geographical variation of risks in such small administrative units [4-6].

Recently, within MEDEA, Borrell et al. [7] have explored the relationship between deprivation and several causes of mortality in this group of eleven Spanish cities. More precisely, ten diseases (among the main causes of mortality) were analysed for men and another ten were analysed for women. An ecological mixed effect spatial regression model was carried out independently for each combination of cause of mortality and city. The units of study for these analyses were also census tracts.

The study by Borrell et al. determined the corresponding relative risk associated with deprivation for each city and cause of mortality. However, it did not determine a common general relative risk that could be generalized to any other city with similar features. If those city-specific relative risks had been considered as a sample of values (random effects) instead of specific values corresponding to specific cities, they could have been used to generalize the findings of the study to any other hypothetical big city, making the new results more general and valuable. In this sense, a pooled cross-sectional analysis considering these relative risks altogether could be carried out to generalize the aforementioned deprivation-mortality relationships into a wider context. Moreover, the estimates from different cities would pool their information, providing improved estimates of the deprivation-mortality relationship for every city. Finally, a joint study for all the cities would allow us to assess whether the size of the cities analysed could be related to the magnitude of the relationships found.

The objectives of our study may be summarized as: (1) to find, whenever possible, a general relationship between deprivation and mortality for each one of the causes of mortality studied, pooling the information from all the cities; (2) to determine whether the deprivation-mortality relationship was just the same or, on the contrary, varied between cities; (3) to estimate (with the corresponding uncertainty measures) the deprivation-mortality relationship for another comparable city not included in the analysis.

## Methods

One of the main results of the MEDEA project was to find the relationship between deprivation and mortality for every cause and city studied. The methodology of the study was fully described by Borrell et al. [7]. Briefly, the population of study consisted of those people residing in the eleven mentioned cities during the period 1996–2003. Mortality data were drawn from the mortality registries of the corresponding regional governments or from the city mortality registry in the case of Barcelona. Mortality was coded following the ICD-9 classification from 1996 to 1999 and ICD-10 from 2000 to 2003.

The data used in this study were drawn directly from Borrell et al. [7]. There, the deprivation-mortality relationship was determined by means of a mixed effects Poisson ecological regression model. Two random effects were included in that model to explain the logarithm of the mortality risk for every geographical unit: one of them spatially structured and the other one spatially independent, following the reasoning in Besag et al. [4]. These two terms are expected to model the unexplained variability by a deprivation index, which is also included in the model by means of a linear term. This deprivation index was derived from a Principal Components Analysis of five socioeconomic indicators (Unemployment; Low educational level; Low educational level in young people; Manual workers; Temporary workers) and was included in the model as a deprivation measure [8]. As the main result of those ecological regression analyses, the relative risk of mortality corresponding to the census tract in the 95th quantile of deprivation (highest deprivation) was determined and compared to that in the 5th quantile (lowest deprivation) for every city (Tables three and four in Borrell et al. [7]). These relative risks are intended to be measures of the impact of deprivation on the different causes of mortality for every city and from now on we will call them “the relative risks”, or simply RR. Relative risk values higher than one suggest worse mortality levels for the most deprived census tracts, while relative risks lower than one indicate worse mortality figures for the least deprived census tracts. Credible intervals of 95% were also computed for RR for each city and cause of mortality in order to assess whether they are statistically different from one or not.

Independent meta-analysis studies have been carried out for every cause of death in our study. In those meta-analyses the logarithms of the relative risks (logRR) have been modelled instead of the relative risks themselves. The reason for working with the logarithms is that these have symmetrical and Normal-shaped posterior distributions; on the other hand, the relative risks have asymmetrical right-tailed posterior distributions. That is, the logarithm of the relative risks fits the Normal assumption that has been assumed in the meta-analysis models much better.

As a measure of uncertainty for every one of the former logRR, we have estimated their standard deviations by means of the length of their 95% credible interval (in logarithmic scale) divided by 2*1.96. This last factor corresponds to the relationship between standard deviations and 95% Confidence intervals under the Normal assumption.

Four different models have been fitted for every cause of death in order to assess which of them best fits the logRR of the different cities. The first proposal models the logRR for all the cities by means of a single common value. This proposal will be referred to as the *Fixed Effect Model* and it assumes the effect of deprivation to be exactly the same for all the cities. In the second one an extension of the Fixed Effect Model is proposed assuming, regardless of the common value for all the cities, the existence of heterogeneity (Gaussian random effect) in the logRR among cities. This proposal will be referred to as the *Random Effects Model*. Due to the quite different population sizes of the cities in the study, and anticipating that this factor could have an influence on the relationships that we are trying to describe, another extension of the Fixed Effect Model has been considered as a third modelling alternative. This time it is assumed that the logRR is a linear function of the population of the cities (obtained from the 2001 Census of the Spanish National Institute of Statistics). Therefore this model proposes the effect of deprivation to be a function of the dimension of the cities instead of being exactly equal for all of them. This model will be referred to as the *Population Regression Model*. Last, the fourth alternative merges both the second and third models. This new proposal considers the Population Regression Model just mentioned, but it also includes a random effect assuming heterogeneity among cities. This last alternative will be called the *Mixed Regression Model* and it considers that the deprivation-mortality relationship varies across cities depending on their sizes and some other unknown factors.

The logarithm of the population of the cities will be included as a covariate in the regression models (third and fourth model), instead of the corresponding populations without any further transformation. This way we will avoid those bigger cities having an outlying effect on the results of these models. Moreover, it is more reasonable to expect that a change of 100,000, inhabitants in the smallest cities in the study (with about 150,000 people) will have much more impact than a similar change in the mortality of the biggest cities (with about three million people). Therefore, the inclusion of the population effect as a relative effect instead of an absolute one seems more appropriate, which justifies the inclusion of the population in a logarithmic scale to assess the effect of the size of the cities. Finally, to make the interpretation of the results easier, the logarithm to base 2 of the population of every city will be considered, as in that case a difference of one unit for that variable has a clear meaning: the size of the corresponding population has been doubled.

The four aforementioned meta-analysis models will be formulated from a Bayesian point of view. Flat non-informative prior distributions will be proposed for all the fixed effects in the models. Gaussian random effects will be used to model heterogeneity among cities with a vague uniform prior distribution for their standard deviations. Inference will be carried out by means of WinBUGS 1.4.3 and R 2.10.1, making use of the R2WinBUGS package to connect both tools.

In order to determine the most appropriate meta-analysis model describing the logRR for every cause of death, we will make use of the DIC model selection criteria [9]. The lowest values of DIC will point to the most appropriate models in predictive terms. One of the main problems of this criterion is that it shows some variability due to the simulation-based inference process. To assess that variability when determining the lowest DIC value, each of the four models will be fitted 100 times and the model with the lowest DIC will be determined for all of them. This will allow us to calculate the proportion of times that the DIC of each model is the lowest one as a reliability measure of the model selection made.

## Results

**Figure 1:**
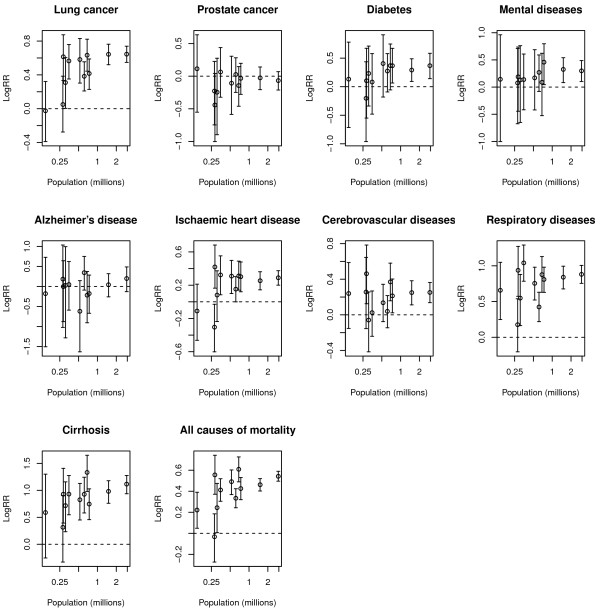
**Relationship between population size and log-relative risk for deprivation for the elven cities studied. **Men.

**Figure 2:**
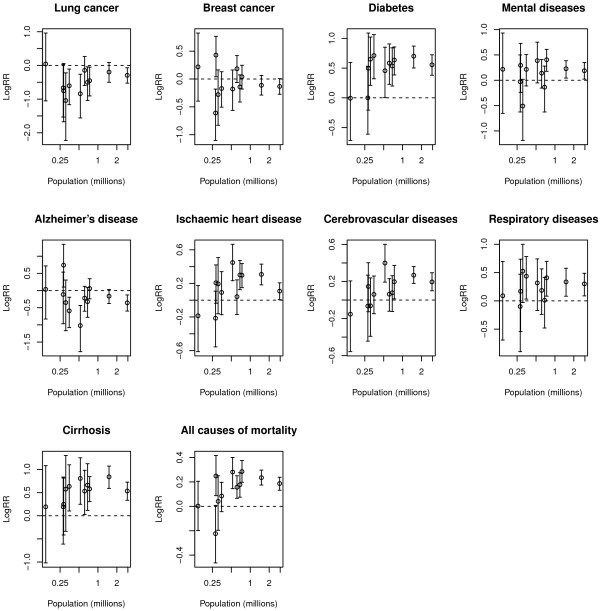
**Relationship between population size and log-relative risk for deprivation for the eleven cities studied.** Women.

Figures 1 and 2 show, for men and women respectively, the relationship between the population of every city in millions of people and the logRR posterior means with their credible interval for every cause of death studied. It is worth recalling that positive values of the log-relative risk indicate worse mortality values for those regions with more deprivation, and conversely negative values indicate worse mortality values for less deprived regions. Different patterns can be appreciated in those figures. For example, on the one hand we find relationships without either a marked trend or substantial variability among cities (for example, prostate cancer in men). On the contrary, we can also find some causes showing these two sources of variability (such as lung cancer in males). Moreover, it can be appreciated that, in general, for most causes of death deprivation increases mortality (logRRs higher than 0) and this effect in general seems more evident in bigger cities for which the variability of that relationship is lower.

In order to select which of the four models considered is more appropriate for every cause of death, on a more objective and non-visual basis, Table 1 shows the DIC for every model and the proportion of times, out of 100 replications, that each of them has attained the lowest DIC for the four different models considered (in brackets). For every cause of death and sex, the model with the lowest DIC (the best according to this criterion) appears in bold in Table 1.

**Table 1:** DIC criterion for the four models considered and proportion of replications that each model has been the one with the lowest DIC (in brackets)

**Causeof death**	**Sex**	**DIC**	**DIC**	**DIC**	**DIC**
		**Model 1: Fixed effect model**	**Model 2: Random effects model**	**Model 3: Regression model**	**Model 4: Mixed regression model**
Lung cancer	Men	36.13 (0)	21.68 (0)	22.51 (0)	**20.64 (1)**
Lung cancer	Women	11.65 (0)	13.13 (0)	**11.28 (1)**	12.95 (0)
Prostate cancer	Men	**6.59 (1)**	8.58 (0)	8.53 (0)	10.45 (0)
Breast cancer	Women	23.42(0)	**20.85 (1)**	23.96 (0)	21.43 (0)
Diabetes	Men	**6.12 (1)**	8.08 (0)	6.71 (0)	8.65 (0)
Diabetes	Women	**12.01 (1)**	13.70 (0)	13.12 (0)	14.52 (0)
Mental illnesses	Men	**4.59 (1)**	6.55 (0)	5.96 (0)	7.90 (0)
Mental illnesses	Women	**14.71 (1)**	15.96 (0)	16.63 (0)	17.38 (0)
Alzheimer’s disease	Men	**8.73 (1)**	10.39 (0)	10.04 (0)	11.71 (0)
Alzheimer’s disease	Women	25.19 (0)	**21.24 (0.62)**	26.16 (0)	21.34 (0.38)
Ischaemic heart disease	Men	27.52 (0)	23.17 (0)	25.47 (0)	**22.26 (1)**
Ischaemic heart disease	Women	28.84 (0)	20.85 (0)	30.84 (0)	**20.11(1)**
Cerebrovascular diseases	Men	**15.73 (1)**	16.35 (0)	16.29 (0)	17.17 (0)
Cerebrovascular diseases	Women	21.26 (0)	18.75 (0)	18.24 (0)	**17.30 (1)**
Respiratory diseases	Men	34.21 (0)	**20.88 (0.89)**	31.45 (0)	21.19 (0.11)
Respiratory diseases	Women	**7.50 (1)**	9.42 (0)	9.44 (0)	11.30 (0)
Cirrhosis	Men	18.92 (0)	17.72 (0)	**14.88 (1)**	15.91 (0)
Cirrhosis	Women	**10.10 (1)**	11.36 (0)	11.76 (0)	12.52 (0)
All causes of mortality	Men	57.89 (0)	**22.47 (0.72)**	37.09 (0)	22.65 (0.28)
All causes of mortality	Women	31.30 (0)	23.56 (0)	30.24 (0)	**21.94 (1)**

In general it can be appreciated that the model with the lowest DIC is usually consistent over the different replications carried out, therefore uncertainty in the determination of the model with the lowest DIC is usually low. The only causes of death for which our replications do not unequivocally determine a single model are Alzheimer’s disease for women, respiratory diseases for men, and ‘all causes of mortality’ for men. For all these, the lowest DIC is undetermined between the Random Effects Model and the Mixed Regression Model, although for all of them the Random Effects Model seems to be the most reasonable alternative.

Examining Table 1, it can also be appreciated that the relationship between deprivation and mortality does not change across different cities for the following causes of death: prostate cancer, diabetes (both sexes), mental illnesses (both sexes), alzheimer’s disease (men), cerebrovascular diseases (men), respiratory diseases (women) and cirrhosis (women). That is, for all these causes of death a single common log-relative risk would be reasonable for all the cities either because there are no differences between cities or because such differences are too small to be found by the study. Table 2 shows the estimated common log-relative risks (with their 95% credible intervals), pooling information from all the studied cities.

**Table 2:** Main results for those causes of death best fitted with the fixed effect model

**Cause of death**	**Sex**	**Common log-relative risk for deprivation**
Prostate cancer	Men	-0.059 [-0.140, 0.022]
Diabetes	Men	0.298 [0.194, 0.407]
Diabetes	Women	0.586 [0.494, 0.678]
Mental diseases	Men	0.282 [0.177, 0.389]
Mental diseases	Women	0.213 [0.129, 0.296]
Alzheimer’s disease	Men	0.065 [-0.088, 0.215]
Cerebrovascular diseases	Men	0.209 [0.153, 0.267]
Respiratory diseases	Women	0.300 [0.192, 0.404]
Cirrhosis	Women	0.613 [0.500, 0.721]

We find evidence of association (the corresponding 95% credible interval does not contain zero) between deprivation and mortality for all these causes of death, except for prostate cancer and Alzheimer’s disease (men). Moreover, all the associations found go in the same direction: an association is found between higher levels of deprivation and higher mortality risks. For all these causes the model selected assumes a common relationship between deprivation and mortality for all the cities and therefore observed differences between cities should be attributed to random variability. We would also point out that the corresponding credible intervals for the log-relative risks in our analysis are much narrower than those intervals for the cities individually, as the former pool the information on the log-relative risks of all the cities.

For the second model, Table 3 indicates the group of causes for which the deprivation-mortality relationship has been found to vary around a single value across cities, but with relevant differences among them. These differences may not be attributed to the size of the cities either because that relationship does not exist or the differences are too small to be found by the study. These causes are the following: breast cancer (women), Alzheimer’s disease (women), respiratory diseases (men) and ‘all causes of mortality’ (men). Table 3 shows the expected logRR for every cause of death and the standard deviation of the random effect modelling the heterogeneity of the logRRs.

**Table 3:** Main results for those causes of death best fitted with the random effects model

**Cause of death**	**Sex**	**Common log-relative risk for deprivation**	**Standard deviation of differences between cities in log-relative risk**
Breast cancer	Women	-0.062 [-0.222, 0.091]	0.167
Alzheimer’s disease	Women	-0.209 [-0.486, 0.028]	0.297
Respiratory diseases	Men	0.745 [0.579, 0.891]	0.217
All causes of mortality	Men	0.407 [0.283, 0.516]	0.171

Evidence of a general association between deprivation and mortality has been found for respiratory diseases (men) and ‘all causes of mortality’ (men) for all the cities altogether. On the other hand, that relationship has not been found for breast cancer and Alzheimer’s disease (women). Moreover, some variability in the results has been found among the cities, that variability being higher for Alzheimer’s disease (women) and lower for breast cancer (women). As a consequence, for these causes, deprivation might be either a protective factor or a risk factor, depending on the city under study. Specific logRR estimates for each city on these causes of death (results not shown) are close to those outlined by Borrell et al. [7]. Nonetheless, we now have an estimate available of the pooled relative risk across the cities and its uncertainty, which was previously unknown.

From the common expected log-relative risk for all the cities and the standard deviation of the random effects modelling heterogeneity among the cities, we can derive the predictive distribution of the log-relative risk for any new city not included in the analysis. Therefore, the resulting predictive posterior means and the 95% predictive credible intervals would be the following: breast Cancer -0.065 [-0.534, 0.349] Alzheimer’s disease -0.213 [-1.09, 0.504], respiratory diseases 0.752 [0.236, 1.258], and ‘all causes of mortality’ 0.423 [0.00, 0.517]. Thus, for any hypothetical new city we would expect to find either a positive or negative association between deprivation and mortality for breast cancer and Alzheimer’s disease (women), that is, deprivation could be either a risk factor or a protective factor for these causes, depending on the city. On the contrary, for respiratory diseases (men) and ‘all causes of mortality’ (men), we would expect deprivation to increase the risks for any other hypothetical new city, although with different strengths for different cities.

Third, in Table 1 the relationship between deprivation and mortality for lung cancer (women) and cirrhosis (men) is only associated with the size of the cities studied. No variability across cities is found in this case either because it does not exist or it is too small to be found by the study. Table 4 shows the logRR for a city of one million people and the regression coefficient associated with the size of the cities. That coefficient describes the effect on the logRR of doubling the population of any city, that is, if positive, the most deprived areas will have worse mortality results in larger cities than in smaller cities.

**Table 4:** Main results for causes of death best fitted with the regression model using population as a covariate

**Cause of death**	**Sex**	**Log-relative risk for deprivation (city of 1 million inhabitants)**	**Effect of city size on the log-relative risk for deprivation**
Lung cancer	Women	-0.380 [-0.507, -0.248]	0.086 [-0.022, 0.200]
Cirrhosis	Men	0.965 [0.873, 1.059]	0.096 [0.019, 0.170]

Significant associations have been found for lung cancer (women) and cirrhosis (men) as causes of death in cities of one million people. Nevertheless, those associations go in opposing directions: Lung cancer mortality for women is lower in the most deprived census tracts while cirrhosis mortality for men is higher for those areas with the highest deprivation. However, the effect of increasing the size of the city goes in the same direction: for both causes the most deprived regions come out worse in larger cities. The association found between deprivation and mortality for lung cancer in women vanishes for larger cities while for cirrhosis in men that same association increases with the size of the city. Yet deprivation has been found to be a protective factor for lung cancer even for Madrid (the largest city in the analysis, with nearly three million people). This means that we would have to consider an even larger city in order to find no relationship between deprivation and this cause of mortality. Similarly, deprivation has been found to be risk factor for cirrhosis in men even for Castellón (the smallest city, with around 150,000 inhabitants). Therefore, for all the cities studied, significant associations have been found between deprivation and these two causes of mortality, although its strength depends on the size of the city.

Finally, the deprivation-mortality relationship has been found to depend on both the size of the cities and other unknown factors modelled as random effects for lung cancer mortality (men), ischaemic heart disease (both sexes), cerebrovascular diseases (women) and ‘all causes of mortality’ (women). Table 5 shows the expected logRR for these causes of death for a city of one million inhabitants, the regression coefficients associated with the size of the cities and the standard deviation of the heterogeneity of logRRs for the cities in the analysis.

**Table 5:** Main results for causes of death best fitted with the mixed regression model using population as a covariate.

**Cause of death**	**Sex**	**Log-relative risk for deprivation (city of 1 million inhabitants)**	**Effect of city size on the log-relative risk for deprivation**	**Standard deviation of differences between cities in log-relative risk**
Lung cancer	Men	0.550 [0.427,0.690]	0.106 [0.018,0.213]	0.126
Ischaemic heart disease	Men	0.253 [0.111,0.411]	0.064 [-0.031,0.178]	0.146
Ischaemic heart disease	Women	0.209 [0.055,0.364]	0.050 [-0.061,0.174]	0.174
Cerebrovascular diseases	Women	0.175 [0.075,0.279]	0.060 [-0.011,0.142]	0.096
All causes of mortality	Women	0.193 [0.101,0.288]	0.048 [-0.018,0.122]	0.102

Significant associations have been found between deprivation and mortality for all these causes of death for a hypothetical city of one million inhabitants. Moreover, all these associations go in the same direction: most deprived areas have worse mortality levels. On the other hand, the size of the cities has the same effect for all these causes of death, with deprivation having a greater (risk) effect in larger cities. Lastly, standard deviations of the random effects vary from 0.096 to 0.174, depending on the cause of death.

For this model it is not possible to derive the predictive distribution of the deprivation effect for a generic hypothetical city as that distribution would depend on the size of the city. Nevertheless, for illustrative purposes, for lung cancer (men) the estimate of the common logRR for a city of one million people is 4.36 times larger than the standard deviation of heterogeneity among cities. Therefore, for a hypothetical new city of this size, we would expect to find a positive association between deprivation and this cause of mortality. On the other hand, for Ischaemic heart disease (men), cerebrovascular diseases (women) and ‘all causes of mortality’ (women) the previous relationship between the logRR and the standard deviation falls to 1.73, 1.82 and 1.90, respectively. Thus, for these causes of death in a city of one million people, we would also expect deprivation to increase the risk of mortality in general, but less so than for lung cancer. Obviously, for larger cities it would be more likely to find a positive association between these two factors. Last, for ischaemic heart disease in women, the former relationship falls to 1.20, therefore for any new hypothetical city (of one million inhabitants) deprivation can in general either be a protective factor or risk factor.

For this model it could also be of interest to compare the proportion of variance of the differences between cities explained by their size and that explained by the random effect and attributable to other unknown factors. In that case we find the proportion of variance of the differences among cities explained by their size to be 51.2% for lung cancer for men, 22.2% for ischaemic heart disease for men, 10.9% for ischaemic heart disease for women, 36.6% for cerebrovascular diseases for women and 24.7% for ‘all causes of mortality’ for women. As a consequence, it can be seen that in general the variance explained by the unknown factors is larger than that attributable to the size of the cities. For these causes of death there are other factors remaining that modify the deprivation-mortality relationship and these factors have an important influence on the differences between cities.

## Discussion

Deprivation has already been shown to be an important factor for mortality [10-14]. Our purpose in this study has been to show the influence of deprivation on mortality in several Spanish cities of different sizes, trying to extract the common features from all of them that explain the general relationship between deprivation and mortality in urban settings. The causes of death studied were the same as those analysed in the MEDEA project and they are the main causes of mortality in Spain. The influence of deprivation on mortality for every cause has already been studied independently in each specific city [7]. The main contribution of our study is to make a joint view of the results in Borrell et al. [7], shedding some light on the existence (or absence) of heterogeneity among cities in the deprivation-mortality relationships studied.

In general, when a relationship between mortality and deprivation has been determined, this usually goes in the same direction: areas with higher deprivation show higher risks of mortality. Moreover, when the size of the cities studied has been found to have a modifying role on the former associations, the deprivation-mortality relationship is even more evident in the most populated cities. That is, when it plays a role, the size of the city increases these inequalities, making deprivation-related differences between areas more extreme. This agrees with Batty [15], who points out that bigger cities can show larger inequalities as they are more complex, poorly-integrated conurbations.

We used several models to fit the data and we found that a wide group of causes are explained better with fixed effect models. This means that for these causes (11 out of 20), once the logRRs have been explained, either as a common value or as a function of the size of city, heterogeneity among the relative risks of different cities can be discarded. Therefore, for these causes, logRRs may be reasonably explained with just an intercept and (sometimes) the effect of population. No more terms are needed to explain these logRRs. For these settings where heterogeneity among the relative risks of different cities is low, the benefits of performing this kind of pooled cross-sectional analyses, merging information from all the cities, seem evident.

On the other hand, for 9 out of 20 causes, random effects are needed to explain the variability of risks among the cities appropriately. The inclusion of these random effects denotes the existence of covariates (apart from population) modifying the risks and these are not considered in the analysis. The determination of the covariates responsible for that variability could help to reduce deprivation-related inequalities.

One interesting result of our study is to determine for which causes the deprivation-mortality relationship is constant across cities and for which causes it is not. This result could be a guide for future studies as the causes in this second group should not be studied for just one single city, as the results of that study would show a city-specific component precluding their generalization into a wider context. Joint analyses of several cities, such as the one carried out in this paper, allow us to derive results for urban areas in general (avoiding city-specific conclusions) and to quantify the variability among cities of the effect of deprivation on mortality.

We found some causes that showed variations in the deprivation-mortality relationship among cities. Of these, breast cancer and Alzheimer’s disease in women showed no evidence of an association with deprivation. For breast cancer, there is a controversy about that association in the literature: some studies [16] have shown differences in mortality trends related to deprivation and conversely others have not [17]. Our results are in agreement with that controversy, as we conclude that that association may vary among cities (therefore, conclusions may vary easily among studies). Our pooled logRR is quite close to zero and heterogeneity makes it perfectly possible to find cities either with a deprivation-mortality association (even of different signs) or cities without one. However, as far as we know, no relationship has been found between deprivation and Alzheimer’s disease mortality. In the light of our results, this relationship will vary among cities, making deprivation either a risk or a protective factor, depending on the city.

In our study, we found evidence of an association with deprivation that was not attributable to population size either for respiratory diseases or for ‘all causes of mortality’ in men. The link between deprivation and mortality from respiratory diseases has been consistently found for men and is linked to smoking, housing and the quality of air. These factors could vary for cities in Spain. A future study including these and additional covariates for a greater number of cities could shed some light on the factors which explain the differences among cities found in this study.

Differences in the deprivation-mortality relationship among cities have already been reported in the United Kingdom [18,19]. It is argued that there are more aspects beyond deprivation that act in different ways in different cities [20]. This factor could also be an explanation for the results in our study: there may be other factors modifying the effect of deprivation in every city. That is, heterogeneity among cities could be explained as an interaction between deprivation and certain other varying factors. The exploration of this issue would require a pooled cross-sectional study considering other factors (not only deprivation) and their interactions as covariates explaining mortality.

### Limitations and strengths

The main limitation of this study comes from its ecological character. Individual conclusions should not be drawn from this study to avoid the effect of the ecological fallacy. Nevertheless, the presence of ecological bias in our results may be lessened by the small size of the units of study (census tracts contain, on average, 1,212 people) [21]. A second limitation of this study is the change in coding from the ninth to the tenth revision of the ICD in 1999. Nevertheless, a prior study at national level which analysed agreement between ICD-9 and ICD-10 for the leading causes of death found that differences between classifications were only minor (under 3%) [22].

Another limitation of the study is the exclusion of covariates other than the population sizes. We are conscious that other variables apart from population sizes could explain the heterogeneity among cities, for instance, urban density, average annual income in the cities, geographical location, etc. Nevertheless, as our study is limited to eleven cities, it is not possible to discern which of these or other factors are responsible for the variability found. We have limited our exploration to the effect of population sizes due to the large variability shown by the cities in this aspect and we have found that for nine causes (out of 20) population size (partially or fully) explained the differences. Therefore, the inclusion of this variable seems fully justified. Moreover, we have also found that eleven (out of 20) causes were appropriately modelled without the inclusion of any random effect. That is, no further variability (except for that explained by the population size of the cities) is required to describe heterogeneity among cities properly. For all those cases, it is not expected that the inclusion of new variables in the models would improve the explanation of the deprivation-mortality relationship among cities.

Borrell et al. [7] originally considered logRRs to be a linear function of the deprivation index. This rigid parametric relationship could be considered a limitation of the study. Nevertheless, we would not expect to find highly non-linear deprivation-mortality relationships as, if deprivation were found to be influencing mortality, it would seem reasonable for that relationship to be monotonic (that is, either increasing or decreasing for all their values). In that case, the relationship could be reasonably fitted by a linear trend for most cases. On the other hand, the linear assumption has made it possible to reduce the deprivation-mortality relationship to a single value, making it possible to undertake our study.

Among the main strengths of our study, we would like to stress that, although a meta-analysis approach has been undertaken, it combines results coming from the same source (the MEDEA project), and the methodology used for its analysis has been identical for the different cities. Moreover, we also think it important that we have information available on the deprivation-mortality relationship for several cities since, as appropriately illustrated in this study, the study of that association in a single city could yield misleading conclusions due to the observed heterogeneity among cities. Finally, the method used allows us to obtain a predictive distribution of the log-relative risk for any new city not included in the analysis, favouring the exportation of results to any other hypothetical city of similar characteristics.

## Conclusions

Our study shows that there are important inequalities in mortality due to deprivation in Spanish cities and, for a set of specific causes of mortality; these inequalities are quite similar for all cities. We have also found variations in the association of deprivation-mortality among cities for certain of these causes of death. These differences have sometimes been linked to the size of the cities, or they have sometimes been attributed to other factors such as smoking behaviour, alcohol consumption, environmental causes, etc. In other causes, such as breast cancer or Alzheimer’s disease, the controversy about the attribution of deprivation remains, although it has been partially explained as a consequence of heterogeneity among cities.

## Competing interests

The authors declare that they have no competing interests.

## Authors’ contributions

MAMB conceived the study, participated in the statistical analysis and drafted the manuscript. OZ conceived the study and helped to draft the manuscript. PBR and MMDO participated in the statistical analysis and helped to draft the manuscript. AN and JM helped to acquire the original data and to draft the manuscript. AD, MFDB, AG, CM, IM, PSV, MT helped to acquire the original data and critically revised the manuscript. CB coordinated the study. All authors read and approved the final manuscript.

## Pre-publication history

The pre-publication history for this paper can be accessed here:

http://www.biomedcentral.com/1471-2458/13/480/prepub
